# Monitoring of Fungal Diversity and Microclimate in Nine Different Museum Depots

**DOI:** 10.3390/jof11070478

**Published:** 2025-06-24

**Authors:** Katharina Derksen, Peter Brimblecombe, Guadalupe Piñar, Monika Waldherr, Alexandra Bettina Graf, Pascal Querner, Katja Sterflinger

**Affiliations:** 1 Institute of Natural Sciences and Technology in the Arts (INTK), Academy of Fine Arts Vienna, Augasse 2-6, 1090 Vienna, Austria; g.pinarlarrubia@akbild.ac.at; 2 School of Environmental Sciences, University of East Anglia, Norwich NR4 7TJ, UK; p.brimblecombe@uea.ac.uk; 3 Department of Marine Environment and Engineering, National Sun Yat-sen University, Kaohsiung 80424, Taiwan; 4 Department of Applied Life Sciences, FH Campus Wien, Favoritenstraße 226, 1100 Vienna, Austria; 5 1. Zoology, Natural History Museum Vienna, Burgring 7, 1010 Vienna, Austria; 6 Institute of Zoology, University of Natural Resources and Life Sciences, Gregor-Mendel-Straße 33, 1180 Vienna, Austria

**Keywords:** monitoring, mold, indoor microclimate, metagenomics, museum repositories, Lower Austria, Vienna, cultural heritage, historic collections, biodeterioration risk

## Abstract

Within museum depots, the largest part of all heritage collections is stored. Often, the preservation of highly sensitive objects is an ongoing challenge, as the materials are constantly subjected to and influenced by ever-present environmental factors—above all the surrounding climate and other physicochemical processes. Biological degradation is also a major risk for collections. Fungal infestation poses a particular threat, in many regions increasingly the result of climate change. Models for damage prediction and risk assessment are still underdeveloped and require a more substantial database. Approaching this need, nine museum depots and archives were selected in this study. Two years of monitoring the indoor microclimate with thermohygrometric sensors, investigating fungal abundance and diversity through culture-dependent and -independent (metagenomics) approaches, and the collection of relevant additional information resulted in a vast amount of diverse data. The main fungal genera identified through cultivation were *Cladosporium*, *Penicillium*, *Aspergillus*, *Alternaria* and *Epicoccum*. The cultivation-independent approach identified *Aspergillus*, *Pyronema*, *Penicillium*, *Xenodidymella* and *Blumeria* as the main taxa. Data analyses indicated that key drivers involved in similarities, patterns and differences between the locations were their geographic location, immediate outdoor surroundings and indoor (micro)climatic fluctuations. The study also sheds light on a possible shift in focus when developing strategies for preventing mold growth in collection depots beyond the prevailing path of tightest possible climate control.

## 1. Introduction

Museum repositories and archives are quite particular indoor spaces. They usually house vast numbers of objects—often consisting of hygroscopic or humidity-sensitive, fragile, historic materials—in secluded interiors with very low visitor frequencies and thus little contact with the outside world. Modern depots, but also some historic examples, are designed and purpose-built to create an environment that ensures the highest possible protection and long-term preservation of the valuable collections kept within, focusing mainly on climatic parameters [1,2,3,4].

Climate and especially variations in climate can have strong physical, mechanical or chemical effects on historic artefacts stored indoors, and are already studied in some depth within the field of conservation and heritage science [5,6,7]. Biological deterioration processes also act on materials and have received less detailed attention, especially concerning microorganisms. Among the latter, fungi pose one of the greatest threats, mainly because of their broad, versatile metabolic capabilities and ability to grow in almost any environment—even in comparatively dry indoor spaces such as a museum depot [8,9,10]. Apart from potential health risks, infestations can lead to irreversible destruction of single objects and entire collections and can cause discoloration and direct or indirect structural damage [8,11,12]. Such incidents are usually accompanied by high treatment costs. Certain filamentous fungi, xerophilic and xerotolerant species (often from the genus *Aspergillus* or *Penicillium*), are specially adapted to grow at low water activities and are commonly found in indoor environments. A rising number of case studies report fungal infestations occurring even in controlled conditions of ≤60% relative humidity (RH), but most common climate guidelines for museums and models for indoor mold growth risk do not yet account for these types of fungi [13,14,15,16,17,18].

Climate change adds a further palette of risks. Predicted increases in extreme weather events, rising temperature (T) and humidity changes are likely leading to an increased risk of mold growth [19,20,21,22,23]. Today, climate control systems, installed to ensure the most ideal climate for preserving cultural and natural heritage collections, are reaching their limits in the hot summer periods. Rising costs of installing or upgrading HVAC systems pose questions about future preservation conditions in storage rooms. Most research on filamentous fungi in the museum environment focuses on single events of active infestations, a small number of rooms or short-term monitoring only [24,25,26,27,28,29]. Sustainable solutions for reducing the risk of biological growth need refined models and simulations to better understand how a change in climate affects collections indoors. These require a substantial database on the status quo within museums and archives without active mold growth. The research presented here is part of a multidisciplinary project aimed at creating such a baseline through broad, long-term monitoring approaches and building representative response models [30]. Within this context, the present study aimed to contribute recent and directly comparable field data for further use in building models and to explore the relationship between climatic conditions and fungal diversity within nine different museum depots. At the outset, it was assumed that the microbial community profiles at the different sites would show many similarities (and indicate a possible shared “museum fungal fingerprint”). Another assumption was that they would also differ in some aspects, influenced, among other factors, by the different geographical locations (rural to urban environments) and the interior microclimate.

## 2. Materials and Methods

Over two years, indoor microclimate (T and RH) and fungal loads were monitored within nine different buildings, covering a diverse range of interiors and materials. The focus was placed on collection storage rooms and purpose-built museum depots. A few of them have a tightly controlled indoor climate with HVAC systems, others are only heated in winter, and some are without active climate control (ACC). Biological data were collected through both cultivation-based and molecular methods, to thoroughly investigate the spectrum of potential (future) biodegradation hazards in direct comparison to the microclimatic data recorded in parallel. The broad methodological approach used in this study has already been applied before and described in a previous publication [31]. However, adaptations were made with the application to a larger dataset (comparison of more sites, a longer timeframe and within molecular methods), as shown in the following sections.

### 2.1. Description of Sites

**Figure 1:**
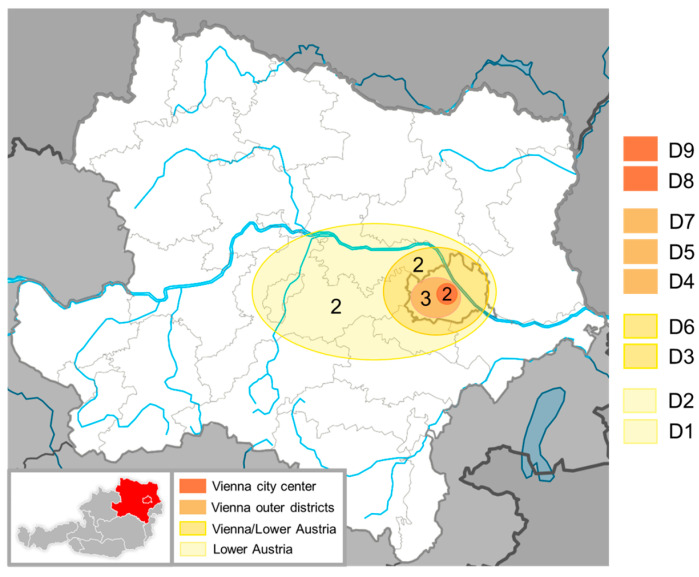
Large map of Lower Austria and Vienna. Orange- to yellow-shaded areas indicate the approximate location of all depots investigated within this study. Numbers written within indicate how many sites are located within each area. The location codes for each depot are listed next to the map, on the right. [Map of Austria: Wikimedia Commons, public domain (https://commons.wikimedia.org/wiki/Category:Blank_maps_of_Austria#/media/File:Map_of_Austria_de.svg (accessed on 4 April 2025)); modified by K.D.; Map of Lower Austria: Rosso Robot, CC BY-SA 3.0 <https://creativecommons.org/licenses/by-sa/3.0>, via Wikimedia Commons (https://commons.wikimedia.org/wiki/File:Austria_Lower_Austria_location_map.svg (accessed on 4 April 2025)); modified by K.D.

Nine sites were chosen for this study, all located in Vienna and Lower Austria; for the purpose of this research, they were anonymized as locations D1–D9. The buildings differ quite strongly in age and structure and are situated in different surroundings, from within the city center to being surrounded by fields. Figure 1 gives an overview of their general location and distance to the city of Vienna. 

They are all either purpose-built as museum repositories or contain rooms that were retrofitted to accommodate various types and sizes of cultural heritage collections. In locations D1, D3, D7 and D9, one room of each building was investigated; in D2, D4, D5 and D6, two separate rooms inside each building were investigated; and in D8, four rooms were investigated. If not specified otherwise, data presented for one location is the combined data from all the rooms of that site. Details on the investigated buildings and rooms are listed in Table S1 (Supplements). Apart from a broad grouping of the nine depots according to geographic location and climate, there are further connecting factors: The three overall most similar buildings (concerning surroundings, building envelope, age, interior, HVAC, and cleanliness) are D2, D3 and D6. D7 and D9 share traits concerning building envelope, age, cleanliness, Compactus® shelving and materials within (mostly paper, as both are archive rooms). The remaining four locations seem less connected, but certain similarities also exist between D8 and D5 (similar building age, furnishing and collection materials) and lastly between D4 and D1 concerning the absence of ACC, large airy rooms and similar collection materials (mainly wood, metal, leather and textile). This is the first study investigating all these sites for a comparison of annual changes in microclimate, fungal loads and diversity.

### 2.2. Indoor and Outdoor Microclimate

**Figure A1:**
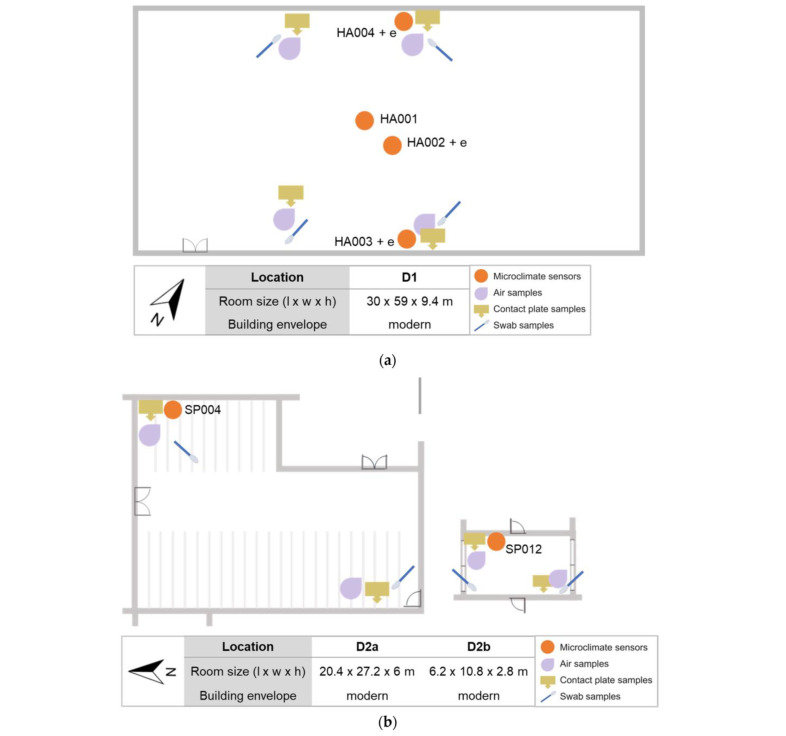

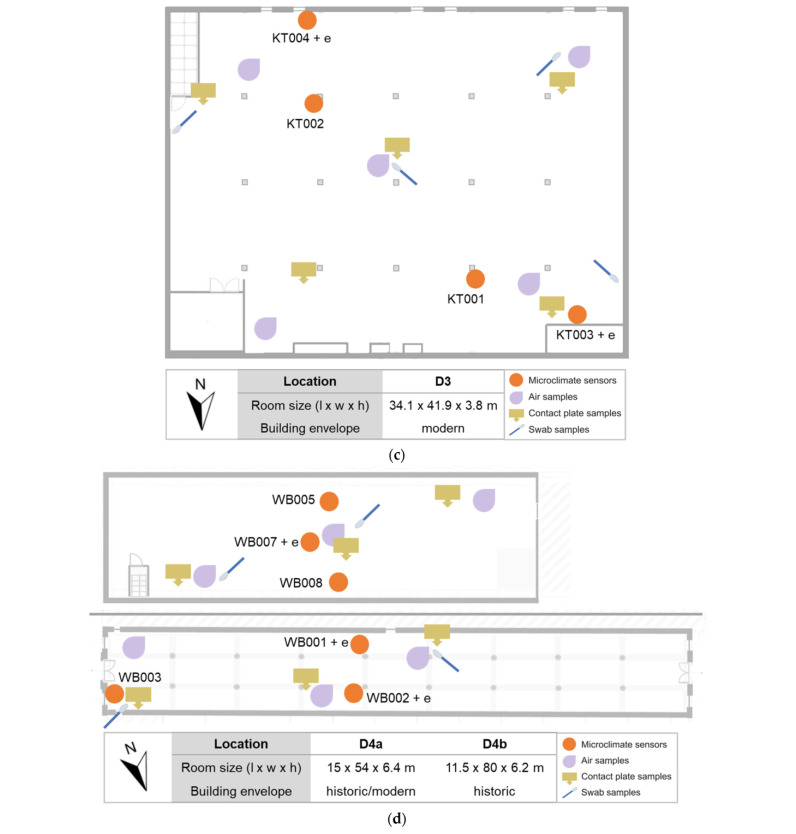

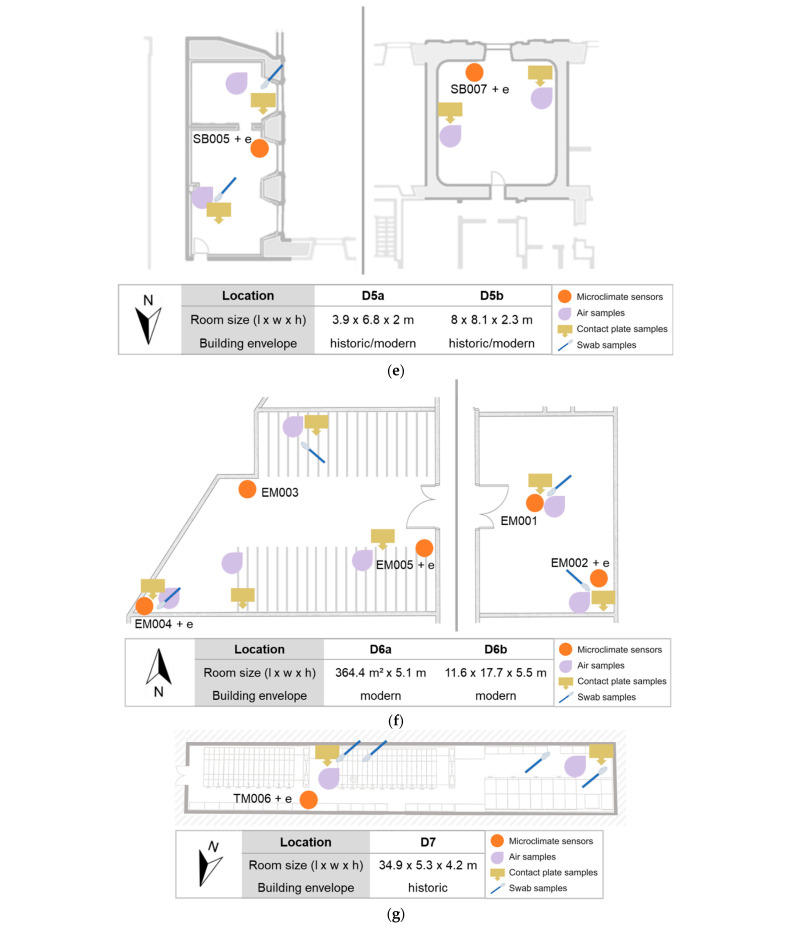

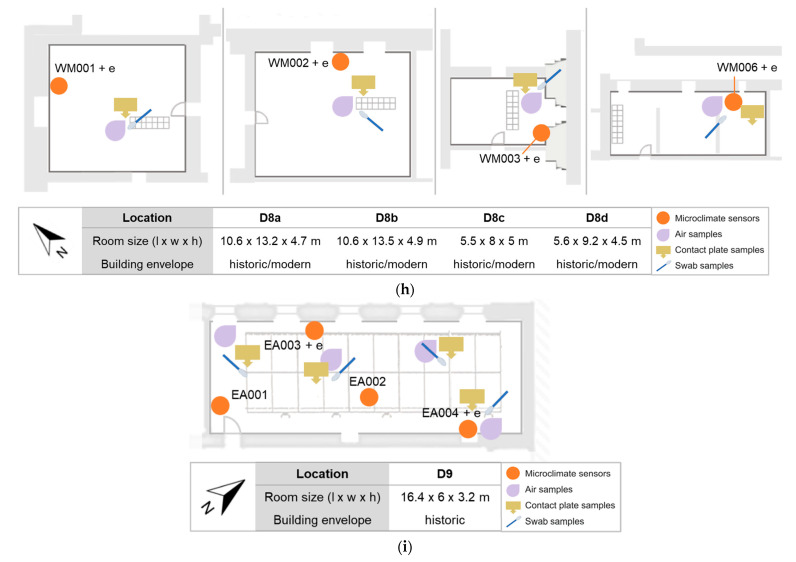
(**a**–**i**) Floor plans of investigated depot rooms: (**a**) Depot D1, (**b**) Depot D2, (**c**) Depot D3, (**d**) Depot D4, (**e**) Depot D5, (**f**) Depot D6, (**g**) Depot D7, (**h**) Depot D8, (**i**) Depot D9. Details on building orientation, age and envelope, room measurements, and locations of thermohygrometric sensors and fungal sampling points are provided. Locations of air and contact plate samples were recurring through the years.

Indoor temperature (T) and relative humidity (RH) data were recorded at 15 min intervals, for an overall duration of 2.5 years, of which the period from September 2021 to September 2023 was the focus for this study. A total of 33 thermohygrometric sensors (Datalogger calibrated by the supplier MostraLog, Long Life for Art, Eichstetten, Germany) were installed throughout the rooms under investigation. They were not placed centrally within the rooms, but in niche spaces, to better represent the relevant microclimates in which biological activity is more likely to occur. In addition to these main sensors, often positioned on top of shelves or close to outer walls, some rooms had further external sensors reaching behind shelves or down to the floor (denoted with an “e” after the sensor number). The placement of sensors within the rooms is shown in Figure A1a–i (Appendix A), and a full list with details on sensors can also be found in the Supplements (Table S1).

For the same time period (September 2021–September 2023) outdoor climate data was compiled for each of the nine locations and downloaded from the GeoSphere Austria (Zentralanstalt für Meteorologie und Geodynamik, ZAMG) website. The geographically nearest and most representative weather stations were chosen for all sites (https://data.hub.geosphere.at/group/stationsdaten, accessed on 24 November 2023; Stations: 5609, 5805, 5904, 5917 and 5925; variables: daily average T, daily average RH).

### 2.3. Microbiological Profiles

The combined approach applied to the analysis and identification of the fungal profiles at each site included three complementary sampling methods: (i) active air sampling with an impactor, (ii) surface sampling with contact plates and (iii) further surface sampling of settled dust using dry sterile cotton swabs for metagenomic analyses.

#### 2.3.1. Sampling

The collection of microbiological samples in the museum depots started in January 2022. Between January 2022 and September 2023, a total of 36 microbiological sampling campaigns took place in the nine locations (4 per location: 1st winter W1, 1st summer S1, 2nd winter W2, 2nd summer S2). Sampling was always performed during the morning, when it was assumed that air was least disturbed. Wherever possible, the sampling points were chosen in proximity to the climate sensors. Figure A1a–i (Appendix A) give a detailed overview of the exact number and locations of sampling points for each room. In total, between four and twelve air samples plus two outdoor reference samples and four to twelve surface samples with contact plates were taken per location during each sampling campaign, depending on room size and complexity. Malt Extract Agar (MEA) and Dichloran Glycerol–Chloramphenicol Agar (DG18) were used for the cultivation of both air (ø 90 mm, device MAS-100 Eco^®^, MBV, Stäfa, Switzerland) and contact plates (ø 55 mm, contact area 16 cm^2^). The air sampling volume was 100 L, and the sampling height was approx. 1.5 m above the floor. Contact plates were applied to surfaces within the rooms such as furnishings, windowpanes, paneling and shelves (for further procedures, see Section 2.3.2). In both seasons of the first sampling year (W1 and S1), additional surface samples were taken from accumulated dust on surfaces inside the rooms (not from collection objects themselves) with sterile cotton swabs. These swab samples (4 per location, each from a surface area of 50 cm^2^) were pooled for each site after DNA extraction in the lab (see Section 2.3.3). A simple visual assessment of the accumulated amount of settled dust on each of the sampled surfaces was recorded concurrently, for which a scale of 1–5 was adopted. The averages at each location were later used for comparisons with data from all surface samples. No case of mold growth or damage was known or detected in any of the rooms at the time of their investigation within this project.

#### 2.3.2. Cultivation Plate Analysis

Viable fungi present in the air and on surfaces were analyzed quantitatively and qualitatively (absolute counts and morphological identification to genus level, in some cases sub-sections) by microscopy (Olympus SZ40, Olympus Optical Co., Ltd., Tokyo, Japan and Leica DM500, Leica Microsystems GmbH, Wetzlar, Germany), after incubation at room temperature for a minimum of seven days (identification literature: [32,33,34,35,36,37]). All counts are given in colony-forming units (CFUs) and expressed as concentrations for air samples (CFUs/m^3^) and contact samples (CFUs/m^2^). For final CFU concentrations, raw counts were multiplied x10 for air (100 L to m^3^) and x625 for surfaces (16 cm^2^ to m^2^).

#### 2.3.3. Metagenomic (Metataxonomic) Analysis

From each swab sample, the total DNA was extracted using the FastDNA™ Spin Kit for Soil and the corresponding protocol (both MP Biomedicals, Eschwege, Germany). The extracted DNA samples of each location (4 swabs per location) were pooled thereafter and concentrated for 1.5 h in a vacuum concentrator (Thermo Scientific Savant SpeedVac DNA 130 Vacuum Concentrator System, Thermo Fisher Scientific, Waltham, MA USA). DNA concentration measurements throughout the entire workflow were performed using a Qbit 2.0 fluorometer (Qbit™ dsDNA HS and BR Assay-Kits, Thermo Fisher Scientific, Waltham, MA USA). The DNA was further amplified by targeted PCR (full ITS region, corresponding to ITS regions 1 and 2, with the 5.8S rRNA gene between them; Primer Sequences 5′-TCCGTAGGTGAACCTGCGG (ITS1, forward) and 5′-TCCTCCGCTTATTGATATGC (ITS4, reverse)) [38,39], as explained below.

A first round of PCR with primers without tails (customized primers ITS1(F) and ITS4(R) (NEB—New England Biolabs, Ipswich, MA, USA) was performed in a BIO-RAD C1000 Touch™ Thermal Cycler to amplify the target sequences, using the LongAmp^®^ Hot Start Taq 2X Master Mix (NEB). The premixed PCR Master Mix 2X was diluted to 1X (final composition 125 units/mL LongAmp^®^ Hot Start Taq DNA Polymerase supplied in a proprietary reaction buffer (pH 9.1), 0.3 mM dNTPs, 60 mM Tris-SO_4_, 20 mM (NH_4_)_2_SO_4_, 2 mM MgSO_4_, 3% Glycerol, 0.06% IGEPAL^®^ CA-630, 0.05% Tween^®^ 20), and 0.5 pmol/µL of each primer (stock: 50 pmol/µL) was added. A total of 4 µL of template DNA was added, and the reaction mix was adjusted to a total volume of 100 µL with nuclease-free H_2_O. The following program was used: 1 min at 94 °C, followed by 35 cycles of 30 s at 94 °C, 30 s at 55 °C, and 50 s at 65 °C, with a final extension step of 5 min at 65 °C.

The Nanopore sequencing platform was selected for the metagenomic analysis (barcoding and library preparation kits, sequencing devices and software were all from Oxford Nanopore Technologies (ONT), Oxford, UK). The new Kit14 chemistry and corresponding protocols (Ligation Sequencing Kit V14 (SQK-LSK114, ONT) and Barcoding Expansion Pack 1-12 (EXP-PBC001, ONT)) were used, as shown in the following.

With a required input amount of 100–200 fmol (corresponding to 50–100 ng for the targeted amplicons) of already-tailed amplicons for the barcoding reaction, an additional PCR run had to precede the next step. This second round of PCR with tailed primers (customized primers from NEB as before, but with added tailing sequences as specified by ONT) was performed to incorporate the tailing/barcode adapter sequences. For this purpose, 0.05 pmol/µL was added to each sample in a reaction volume of 100 µL, from a stock solution of 2.5 pmol/µL, using the same Master Mix at 1X (NEB) and the same Thermocycler program as in the first PCR. The results of both PCR runs were checked by Gel Electrophoresis, using the SYBR™ Safe DNA Gel Stain (Invitrogen™, Thermo Fisher Scientific, Waltham, MA USA) and GelDoc Go Imaging System (Bio-Rad Laboratories, Feldkirchen, Germany) for documentation. Only samples with detectable ITS bands were used to proceed (8 out of 9) with the library preparation.

The remaining steps followed the protocols of the Ligation Sequencing Kit V14 and the Barcoding Expansion Pack 1-12. All samples were adjusted to a concentration of 90–100 ng in 48 µL for the subsequent barcoding reaction. Barcodes 01–08 were assigned to the samples, followed by the pooling of all eight samples for the preparation of a single sequencing library. In the last step, the DNA library was primed and loaded onto a SpotON Flow Cell (FLO-MIN114, ONT), which was previously quality-checked using the MinKNOW™ software 21.11.7 and primed with the addition of 12.5 µL of BSA (Bovine Serum Albumin, Roche CustomBiotech, Mannheim, Germany) to the Flow Cell Priming Mix, as recommended by ONT. Sequencing was performed in a MinION Mk1B device (ONT), with a run duration of 48 h. Base-calling was subsequently performed with dorado (0.9.5) using the SUP model (dna_r10.4.1_e8.2_400bps_sup). All read data were submitted to the NCBI public database (BioProject accession number PRJNA904284).

### 2.4. Data Analysis and Statistics

All outdoor and indoor climate data were imported and prepared and all graphs were created in MS Excel^®^ 2022. For the cultivation data, all information gained from microscopic identification and plate counts was also recorded and further processed in Excel.

Metagenomic data received post-sequencing quality checks, filtering and further treatment up to metataxonomic classification and visualization of results as already described by Derksen et al. [31]. After filtering, the median quality scores (QSs) were between 18.1 and 20.2, representing error rates between 1.55 and 0.95%. Relative abundance cut-offs were set to 0.5% (later presented as a heatmap) and 0.01% (later presented as bar charts) on the shown taxonomic level. Classifications below the threshold were set to “unidentified” or “others”, respectively.

The statistical approaches adopted here needed to recognize that the sample sizes were sometimes small and ordinal in nature with many zeroes, so non-parametric tests were often adopted. We used box and whisker plots to present CFU data, with the box bounded by the 25th and 75th percentiles. The median is denoted by the central line in the box, and the whiskers represent the range of all other points, except those that are deemed as outliers (values beyond 1.5 times the interquartile range). Cluster analysis used the software available at Wessa.net with the Ward method, which gives compact spherical clusters and minimizes variance (https://wessa.net/rwasp_hierarchicalclustering.wasp, last accessed on 6 May 2025). The Shannon Diversity Index (H) was calculated to express entropy (akin to diversity) as *H* = −∑*P*_i_.ln(*P*_i_.), where *P*_i_ is the proportion of the i-th fungus.

## 3. Results

### 3.1. Climate Data—T and RH

Indoor and outdoor climate data were analyzed in direct comparison for all sites, to evaluate external influences on the interior microclimate within the buildings and the impact of ACC systems in some of the rooms. The indoor data shows measurement gaps throughout the second year, due to malfunctioning sensors during these periods. For this reason, some of the following analyses only consider the first monitoring year.

**Figure 2:**
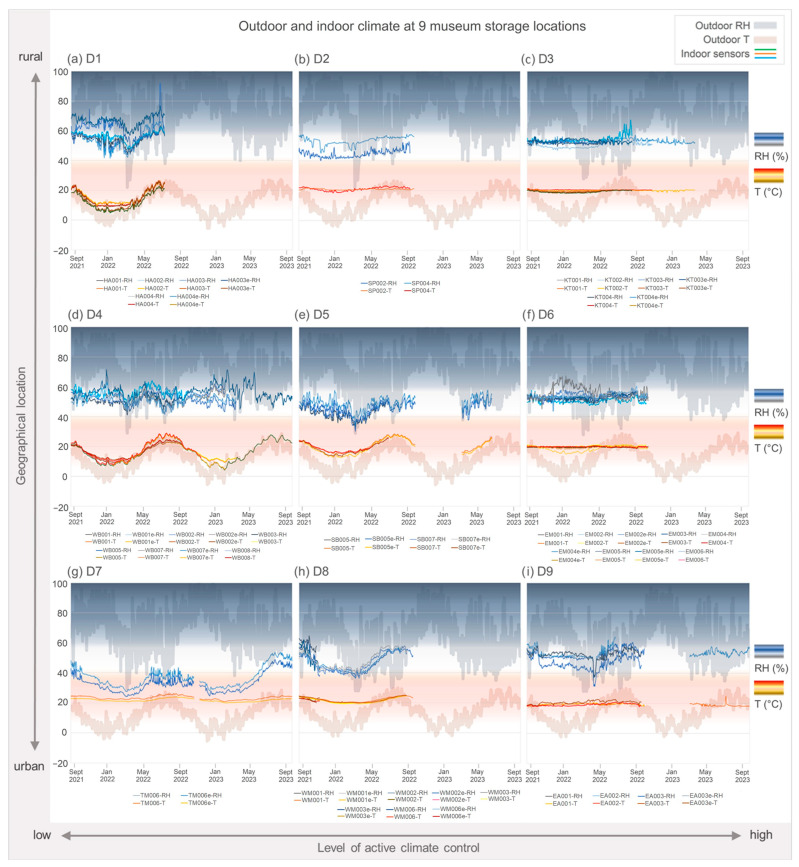
Daily average temperature T (°C; warm colors) and relative humidity RH (%; cool colors), from September 2021 to September 2023 outdoors (low-contrast, greyed-out lines) and indoors (saturated, contrasted lines) at the nine depot locations: D1 (**a**), D2 (**b**), D3 (**c**), D4 (**d**), D5 (**e**), D6 (**f**), D7 (**g**), D8 (**h**) and D9 (**i**). Indoor records (all sensors per location shown) start at the beginning of September 2021 and last until at least the end of September 2022. The blue and red shading in the background highlights regions of T and RH generally referred to as relevant for indoor mold growth risk (stronger color—higher risk).

Daily average values for T and RH (data provided by GeoSphere Austria, see Section 2.2) were plotted together with the indoor data from the thermohygrometric sensors (with daily averages calculated from 15 min interval data). Table 1 summarizes the average annual indoor and outdoor T and RH for all nine locations, for the first year (September 2021–September 2022). Locations D1 and D2 have the lowest annual average outdoor T with the highest RH. D4, D5 and D7 have similar T but are slightly less humid. They are followed by locations D3 and D6, both in a warmer and drier environment. Finally, the average outdoor values for sites D8 and D9 reflect their location close to the generally dry and warm city center of Vienna. In the indoor sensor data (see also Figure 2), the trends follow the geographical location to a certain extent but are strongly influenced by the exact location of the investigated rooms (floor level, immediate surroundings of the building), as well as the level of ACC within the rooms. The latter factor is included in Table 1 for better distinction between the locations.

A clustering analysis (see Figure S1, Supplements), using the average values and standard deviations of indoor T and RH as given in Table 1, confirmed expected similarities between the locations: The four sites without HVAC clustered in two pairs (D1 and D4, D5 and D7). Among those with HVAC, which formed a cluster among themselves, similarities emerged between D2 and D8, followed by D9, then D3 and D6 close to each other.

In Figure 2, the indoor climate measurements from all sensors inside the nine sites (saturated colors, foreground) are shown together with the locations’ outdoor climate (unsaturated, background) for direct comparability. Locations were arranged by, on the one hand, their geographic location and immediate surroundings and, on the other hand, the level of ACC inside the depots. Accordingly, D1 is the most rural location with no ACC system (no heating, cooling, dehumidification or humidification), whereas D9 is in the most urban environment with a high level of climate control (full HVAC).

Temperature profiles follow overall outdoor developments in locations with low ACC, becoming more constant where there is high ACC. The curves also flatten in the more urban locations. D7 and D8 are the only sites where the investigated rooms lie below ground, which is likely to promote stable temperatures. Differences in RH profiles are more complex. D1 had the highest indoor levels of RH, while D7 was the driest of all locations. In some locations, ACC systems seemed to influence the long-term, seasonal fluctuations of RH more than the short-term fluctuations, but the amplitudes of the remaining fluctuations were smaller. A notable point here is that in D8, with full HVAC, RH still fluctuated more strongly over the seasons, albeit with a visible time lag compared to the outdoor curve.

**Figure 3:**
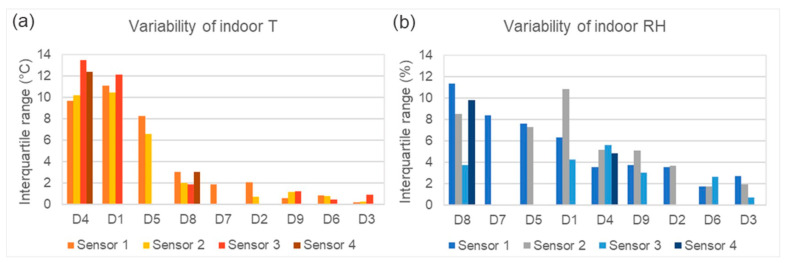
Colum chart showing annual indoor climatic fluctuations expressed as interquartile ranges (IQR) of T and RH in all nine museum depots. Plotted values are IQR of daily values of T (**a**) and RH (**b**), respectively, shown for each sensor in each location (excluding external sensors).

Figure 3 gives an overview of the climatic fluctuations inside all the sites during both monitoring years. The data is split up into single sensors for each location (not including external sensors). The number of sensors per location is shown, as are the differences in variation in indoor T (Figure 3a) and RH (Figure 3b) between the sites. In locations D4, D1 and D5, the indoor average T varied stronger than in all others, by around 2–4 °C, in ranges of around 6–13 °C. D7, located below ground, had the lowest T variation of all sites without ACC. All locations with HVAC systems had visibly lower variations in T (generally <2 °C). The RH variability did not show the same strong difference between the locations with and without HVAC. The highest levels of RH variation (above 6% on average) were observed in D8 (HVAC!), followed by D7, D5 and D1, while all others had average RH variations below 6%.

### 3.2. Microbiological Data

#### 3.2.1. Cultivation Data

**Figure 4:**
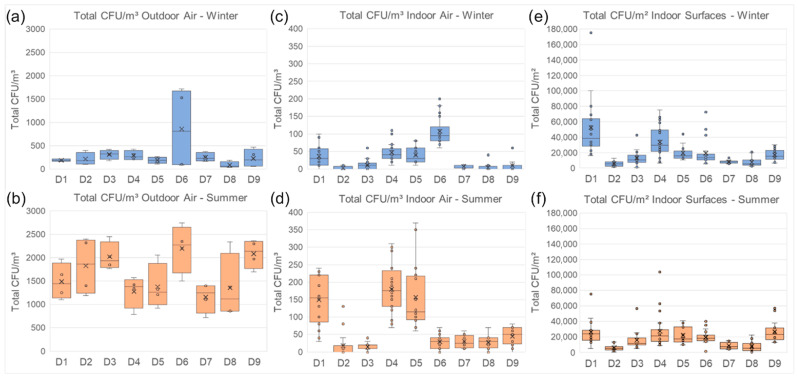
Box–whisker diagram showing the differences in total CFU concentrations of outdoor air (**a**,**b**), indoor air (**c**,**d**) and indoor surfaces (**e**,**f**) between the locations, in winter and in summer, respectively, from both sampling years. Note that different scales were chosen for the diagrams in favor of clearer comparability between sites and seasons rather than sampling type. Data points are shown as circles and “x” denotes the average.

Outdoor air references for the indoor air sampling were taken at every site. An overview of the local weather conditions on each sampling day is provided in the Supplements (Table S2). All cultivation samples from air and surfaces at the sites were collected and incubated as shown in the Methods section. For the quantitative and qualitative analysis that followed, the focus was placed on the most abundant genera and certain taxonomic groups that are also of highest interest for these specific indoor spaces and the collections, as shown below. An overview of the CFU concentrations found in outdoor and indoor air as well as indoor surfaces during all sampling campaigns (2 years) is presented in Figure 4.

In outdoor air, total CFU concentrations were lower in winter than in summer. Winter outdoor air samples were all quite similar, apart from a distinctively higher concentration at D6. The same peak was also visible in the winter indoor air. This was less visible in the summer, but D6 still had the highest median concentration of all outdoor air samples. In a comparison of the different locations, the indoor and outdoor air showed a similar pattern in winter, while the summer indoor air was quite different from the outside. All outdoor summer samples’ median total concentrations varied between 1000 and 2500 CFUs/m^3^. Indoors, all were close to or below 50 CFUs/m^3^, except D1, D4 and D5, which had median values between 100 and 200 CFUs/m^3^.

On indoor surfaces, median values of total CFUs followed almost the same trend between all locations in both seasons. D1 and D4 were the sites with the highest CFUs/m^2^ in winter, with values above 2 × 10^4^, and the lowest values were found in D2 and D8, closely followed by D7 (<1 × 10^4^). In summer, D1 and D4 again showed the highest median concentrations, but also D9 was above 2 × 10^4^ CFUs/m^2^. D2, D8 and D7 still also had the lowest values in summer (<1 × 10^4^ CFUs/m^2^). Summer concentrations on indoor surfaces were overall as low as or lower than those in winter.

The most frequently recurring and abundant organisms identified in all of the cultivation samples were Ascomycota and belong, in descending order of relative abundance, to the genera *Cladosporium*, *Aspergillus*, *Penicillium* and *Alternaria*, followed by *Epicoccum*. Less commonly encountered were fungi of the phylum Mucoromycota and other Ascomycota such as *Fusarium*, *Botrytis*, *Paecilomyces* and *Trichoderma*, but also the basidiomycete *Wallemia*. Rather singular identifications were representatives from the genera *Parengyodontium*, *Neurospora*, *Chaetomium* and *Aureobasidium* and the family Didymellaceae.

**Figure 5:**
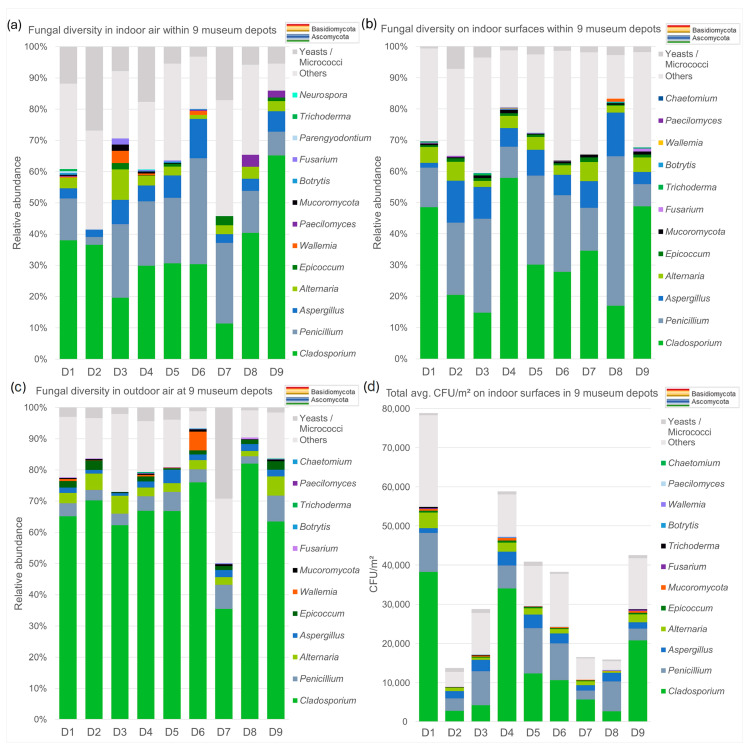
Column charts showing the diversity of genera identified from cultivation samples from indoor air (**a**), outdoor air (**c**) and surfaces (**b**,**d**). Genera are sorted according to relative abundance (total average concentrations in CFUs/m^3^ or m^2^, respectively) across all sites and 2 years. The overall most abundant genus is at the base. “Others” denotes colonies that were not identified down to genus level and sterile mycelia. The color coding distinguishes between the main phyla Ascomycota (cool colors) and Basidiomycota (only *Wallemia*; warm colors); Mucoromycota are shown in black.

Figure 5 presents a comparison of the fungal diversity profiles identified throughout all nine sites over both sampling years. Concerning the air samples (Figure 5a,c), *Cladosporium* was the most abundant genus in outdoor air (almost all above 60%), but much less represented indoors (almost all below 40%), except for D9, where the percentage was nearly the same. Relative fractions of *Alternaria* were quite similar between indoor and outdoor samples, but *Aspergillus* and *Penicillium* (from <5% and 10% outdoor to mostly >5% and around 20% indoor) clearly increased in indoor air, except in D2, where they remained below 5%. Generally, higher relative amounts of micrococci and yeasts were found in indoor air than in outdoor air.

**Figure A2:**
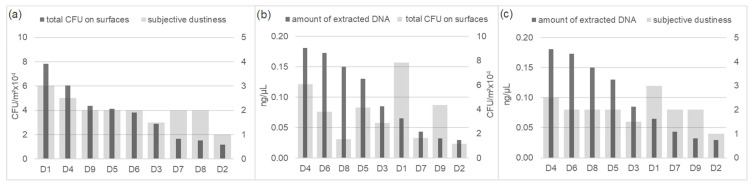
Column charts showing a direct comparison of total CFUs/m^2^ on surfaces, the amounts of extracted DNA and the subjective levels of dustiness (on a scale of 1–5) recorded for each location. Dustiness was first plotted against total CFUs (**a**), and then CFUs were plotted against total extracted DNA (**b**), followed by dustiness plotted against total extracted DNA (**c**).

The contact plate samples revealed the abundance and diversity of culturable fungi on indoor surfaces within all locations (Figure 5b,d). Fractions of the most abundant genera overall match the profiles of the indoor air samples, although Mucoromycota, *Fusarium* and *Trichoderma* were encountered more often on surfaces, and *Chaetomium* were only found on contact plates, while *Parengyodontium* and *Neurospora* occurred only in the air samples. For all sampled surfaces, also a simple visual assessment of the dust amount accumulated on the sampled areas was recorded concurrently. Average totals of CFUs/m^2^ at the locations, as shown in Figure 5d, seem to follow the estimated levels of dustiness (see Figure A2a, Appendix A).

#### 3.2.2. Metagenomic Data

As expected, due to the high level of cleanliness within the depots, the yield of DNA extracted from the swab dust samples was very low. As described above for the cultivation samples from surfaces, a direct comparison of extracted DNA amounts and estimated levels of dustiness on the sampled surfaces was also attempted (see Figure A2b,c, Appendix A). The results showed a connection that was similar, although less clear, to that found for the CFU amounts using contact plates. DNA concentrations measured were between approx. 0.02 and 0.18 ng/µL, with the highest amount recovered from D4, and the lowest from D2.

The two PCR steps, described in Section 2.3.3., nevertheless resulted in sufficient concentrations to prepare DNA libraries for barcoding and sequencing for all locations but for D6. Despite a second run of both PCR reactions with this sample, DNA measurements remained under the detection limit, resulting in the exclusion of this sample from sequencing. From the other eight samples, sequencing generated total reads of 32,289,745, ranging from 860,000 to over 6,750,000 for the single barcodes/sites (see Supplementary Table S3) after quality filtering (adapter/barcodes removed, chimeric sequences split, read ends cropped by 40 bases, length 300–900 bases, Q > 9). The median lengths of the reads used for the assignment were between 440 and 527 bp, in a good range of the targeted amplicon length. The highest amount of read data was recovered from D4, with 6,635,685 assigned reads, followed in descending order by the samples from D3, D7, D8, D5, D2, D1 and D9.

**Figure A3:**
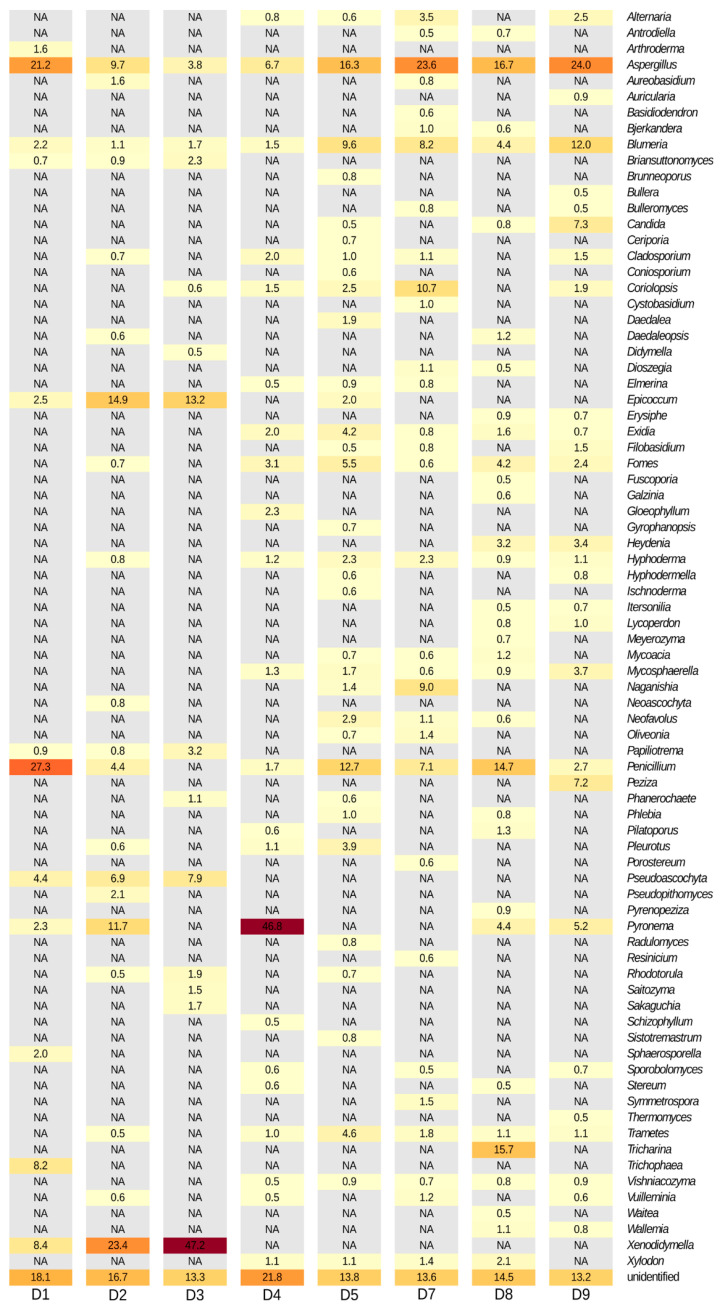
Heatmap displaying the relative abundance (%) of fungal communities (OTUs at genus level, abundance cut-off 0.5%) within the investigated depot rooms, as identified by metagenomic analysis. “Unidentified” denotes classifications identified only to a higher taxonomic level. “NA” denotes classifications below the 0.5% threshold or under the detection limit within that sample.

An overview of the identified taxa at the genus level (based on the relative read abundance for identified genera), as revealed by phylogenetic assignments from DNA sequencing data (relative abundance cut-off 0.5%), is given in Figure A3 (Appendix A). Higher diversities were found at lower abundances, but the cut-off at 0.5 was set in favor of a simplified presentation here. Ascomycota dominated in almost all locations at the phylum level, except for D5 and D7, where Basidiomycota were predominant. The most abundant genera identified by metagenomics over all locations were, in descending order, *Aspergillus* (20%), *Pyronema* (12%), *Penicillium* (12%), *Xenodidymella* (9%), *Blumeria* (7%) and *Epicoccum* (6%). Looking at the single sites, *Aspergillus* and *Penicillium* were the main genera in D1. D2 and D3 were both dominated by *Xenodidymella* and *Epicoccum*. D4 was the only location where *Pyronema* had the highest relative abundance. Depots D5 to D9 generally showed more evenly distributed abundances of genera than the more rural sites. D5 was again dominated by *Aspergillus* and *Penicillium*, D7 by *Aspergillus* and *Coriolopsis* and D8 by *Aspergillus* and *Tricharina.* In D9, *Aspergillus* and *Blumeria* were predominant.

**Figure 6:**
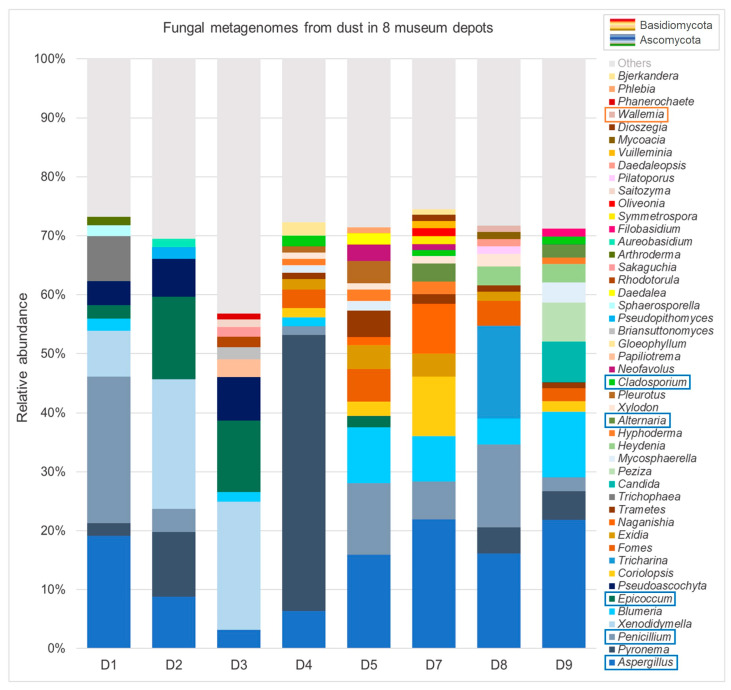
Column chart displaying relative abundance (%) of fungal communities determined from settled dust on indoor surfaces (winter and summer combined for each location) through metagenomics (OTUs; max. genus level resolution; abundance cut-off 0.01), sorted according to abundance across all sites (overall most abundant genus at base, “Others” denotes unidentified OTUs or identifications below the threshold of 0.01). The color coding distinguishes between the main phyla Ascomycota and Basidiomycota. Marked in the list are the main genera also identified through cultivation.

Within Figure 6, the less abundant genera are also listed (>0.01%). It is notable in this comparison that the geographically “intermediate” (between most rural and urban) locations had heightened abundances of Basidiomycota (among them D5 and D7), while Ascomycota prevailed in the metagenomic profiles of all other locations. D1–D3 showed similarities to each other, as did D4–D7, while again D8 and D9 had different “fingerprints”.

**Figure 7:**
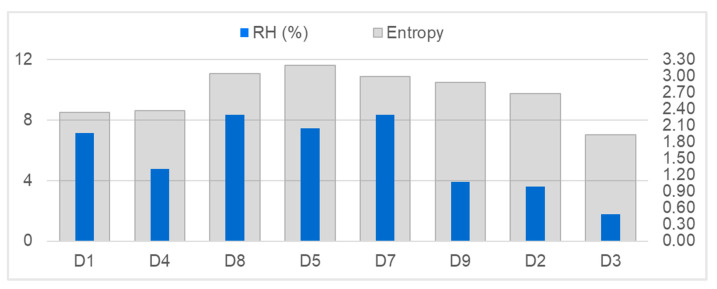
Combined column chart showing the differences in RH variability (average IQR of indoor RH, primary axis) and fungal metagenomic diversity (expressed as entropy, secondary axis) within the museum depots.

As explained in Section 2.4, the entropy (in analogy to the Shannon Index) within each metagenomic sample was adopted as an approximation of the fungal diversity without species-level resolution. The resulting values of entropy for each location are shown in Figure 7, in comparison to the variability of indoor RH (IQR). The three highest values of RH variability coincide with the highest entropy/diversities, found at sites D8, D7 and D5.

## 4. Discussion

Nine museum depots were selected and studied in depth concerning indoor microclimate and fungal diversity as well as abundance, as part of a larger project assessing the impact of climate change on museum pests. A broad but complementary methodological approach combining microclimatic and microbiological monitoring was chosen to create a sound database from which to go deeper into further research directions. All of the studied buildings have a common purpose and use but are set apart by various factors such as their geographical location (rural vs. urban), surroundings, building age and envelope, type of collection materials or the availability of active indoor climate control. At the outset of this study, it was hypothesized that differences in indoor fungal diversity or abundance would be influenced by external factors such as the geographic locations and surroundings, but also the level of control of the interior microclimate. Outcomes seem to be in support of these expectations and are discussed in the following.

### 4.1. Microclimate

Microclimate monitoring data did not reveal many surprises. For most analyses, only the first year of monitoring data was used due to measurement gaps caused by malfunctioning sensors in the second year. The overall development of the indoor climate in the second year of monitoring was assumed to be very similar to the first year, as no changes were made to the building envelopes or climate control systems during that time. As expected, outdoor climate, dictated by the geographic locations, had a strong impact on interior conditions in buildings and rooms without active climate control systems, but less so when there was ACC in place. As was found in our data, ACC systems can result in lower seasonal fluctuations and amplitudes. However, short-term RH variations, in particular, can increase [40]. Interestingly, D7 (with active dehumidification) showed comparatively high RH variability and comparatively low fluctuations in temperature. Looking at the other locations without ACC, D1 and D4 stood out with comparatively low RH fluctuations, even visibly lower than those in D8 (with full HVAC in place). This may be explained by the buffering capacity of the primary collection materials in these rooms [41,42,43]: Mostly, wooden objects are preserved in open storage within D1 and D4. As an archive, D7 showed some of the expected RH-buffering because of the large amount of paper within the room, although closed storage in metal Compactus^®^ Units (mobile aisle shelving) may hinder effective exchange with the hygroscopic material here. A similar situation, albeit less pronounced, was found in D9—again an archive with Compactus^®^ shelving.

### 4.2. Fungal Profiles

Microbiological findings typically followed expected results, such as the general connection of indoor and outdoor air and less clear connection of surface data due to the temporal overlap within settled dust on surfaces, most of which are cleaned at annual intervals. The fungal composition at the genus level shows the most frequent species coming from the taxa *Cladosporium*, *Penicillium*, *Aspergillus* and *Alternaria*, followed by less common *Epicoccum*, Mucoromycota, *Fusarium*, *Botrytis*, *Paecilomyces*, *Trichoderma* and *Wallemia*, so it is very similar to that in other indoor spaces such as living spaces of homes, public libraries, galleries or churches [44,45,46,47,48]. All indoor air CFU concentrations were within normal or even low ranges given in international guidelines (all median CFUs <200/m^3^, all single measurements <400/m^3^) [49,50] and fell within ranges found in other archives and repositories around the globe [51]. No sign of mold growth was observed at any of the locations. Thus, the data is regarded as a representative background baseline.

Looking at the air samples specifically, it was notable that in location D6, winter outdoor and indoor samples showed heightened concentrations. This can most probably be traced to an agricultural holding with animals in the building’s vicinity, and a singular event with high levels of dust being released into the air may have occurred shortly before or coincided with one of the winter sampling campaigns. In all of the sites, median total concentrations for outdoor samples varied between 1000 and 2500 CFUs/m^3^, whereas indoors, three locations clearly stood out in comparison to the rest: only D1, D4 and D5 had median values between 100 and 200 CFUs/m^3^, while the others were all close to or below 50 CFUs/m^3^. Overall, the latter locations seem to show a stronger reduction in the outdoor fungal loads (>100-fold vs only ca. 10-fold), in reaching the indoor depot rooms. However, we still see general similarities in that indoor air patterns follow the outdoor amounts, even in the most modern depots with more or less airtight construction and HVAC systems with built-in air filters (as seen in the example of D6 in the high winter values). Compositions, however, do change, as seen in the strong reduction in *Cladosporium* relative abundances indoors, for instance. The only exception here was D9, where the percentage was nearly the same.

Cultivated surface samples did not show strong differences between the locations in terms of compositions; the relative fractions of the main five genera were very similar to those in air samples, but small differences were visible in the less abundant genera. It should be noted that the overall lower summer values of surface CFU concentrations must be considered an underestimate, due to a flawed batch of commercial media in the first summer sampling campaign (S1). But the fact that it affected all in the same way and that a strong similarity is visible between the seasons at each location led us to believe the results were nevertheless well-suited for relative comparisons between the sites. Despite being a subjective assessment, the simple visual assessment of accumulated amounts of settled dust on all sampled surfaces, recorded concurrently with fungal surface sampling, has been shown to offer a good estimate of actual amounts of dust present on surfaces [52]. Estimated levels of dustiness and CFU concentrations seemed to be indeed connected. Comparisons with extracted DNA amounts revealed a less clear picture but are considered worth exploring further. The generally low amounts of DNA extracted from surface dust can be seen as a sign of a high level of cleanliness within these rooms and correspond to the low amounts of settled dust found (by visual inspection) within all the depots. Despite the low DNA yields obtained from all samples, it was possible to amplify the ITS regions in all of them, with the exception of D6. This might be due to the low performance of PCR in this specific sample.

A number of studies have identified the influences of geographical location on airborne fungal spore compositions and observed gradients between rural and urban areas [53,54]. Haas et al. [55] concluded that, in outdoor air, there was no significant gradient in concentrations of at least the main genera *Cladosporium*, *Penicillium* and *Aspergillus* between rural and urban regions in a different part of Austria, but several other studies seem to disagree on the topic of compositional differences based on a rural–urban gradient alone. Also, our data suggests that only small differences between rural and urban regions were found in cultivation samples. The molecular data, however, seemed to show certain patterns. It is assumed that possible distinctions might only become visible over longer timescales, with metagenomic analyses of settled dust thus opening a “window into the past” by looking further than simple snapshots of fungal profiles found in cultivation samples.

### 4.3. Climate Variability and Microbial Diversity

A previous study by Derksen et al. [31] suggested that the climatic stability of the indoor environments might also influence the biodiversity found within the respective rooms. This was investigated in the nine museum depots, and results again seem to point towards a possible connection. As RH is usually the main determining variable, with variability over time being more influential than steady-state values, a direct comparison between average IQR and calculated entropy (related to diversity) among locations (see Figure 7) showed the highest RH variabilities and highest diversities coinciding in the same three sites.

Most research on outdoor climate variability and the impacts of climate change on fungi focuses on natural habitats and predicts changes such as shifts in or extended fruiting seasons for certain fungi in European countries [56,57,58], as well as changes in metabolic profiles, community structures, abundance and richness [54,59,60]. The strong influence of the outdoors on fungal spore concentrations and diversity indoors is well-known. Despite this, for investigations of indoor environments and the respective detection methods and analyses used, it is equally important for the interpretation of results to consider factors that influence spore viability [61,62]. Certain shifts from outdoor to indoor fungal compositions, which were found in the cultivation samples, may be a product of different survival rates across genera and species [63]. The generally dry interior environments typical of our study are known to favor xerophilic or xerotolerant fungi, as are often found in the genera *Aspergillus* and *Penicillium* [64,65]. All of the main genera identified within this study include species that are commonly encountered in cases of fungal infestations in the museum environment, including *Aspergillus glaucus* and many *Eurotium* species, *A. penicillioides*, *Penicillium chrysogenum*, *P. rubens*, *Cladosporium herbarum*, *C. cladosporioides*, *Alternaria alternata*, *Epicoccum nigrum*, *Wallemia sebi*, *Chaetomium globosum*, *Trichoderma* spp. and *Paecilomyces* spp. [51,66,67,68,69]. All of the above fungi have the potential metabolic capabilities to degrade the various organic materials typical of many historic objects and artworks [8,70,71,72,73]. High abundances of these genera in indoor spaces must therefore always be considered as potential risk factors.

The present study gave unique insights into the fungal communities present in the specific indoor environments of museum depots in northeastern Austria, which has so far not been explored in a comparably extensive way. Thanks to the long-term monitoring of spaces without any current problems of mold growth, it adds a broader context for previous studies on the topic, which most often focused on singular events of infestations. Even though the applied methodology combined culture-dependent as well as -independent methods, which together already allow for a very comprehensive analysis of fungal community composition, there are still ways to improve monitoring approaches in further research. Modifications for future studies to overcome remaining limitations could include (a) the addition of a more specific growth medium for (obligate) xerophilic fungi, which are known to be very relevant for museum environments; (b) working with isolates from cultivation samples for species-level identification; and (c) the use of additional primer sets for reliable species-level resolution in metagenomic analyses.

## 5. Conclusions

The broad and combined methodology of data acquisition applied within this project enabled more of a “bird’s eye view” for the analysis and interpretation of results. In line with the aim of the project, a baseline of microclimatic conditions and fungal profiles was created, which serves interpretations on a more universal level than is possible in singular, site- and case-specific studies. For this long-term and large-scale approach, only locations that did not have any known problems with mold at the time were chosen, and no signs of an active infestation were found throughout the course of this study. The findings point to a possible link between climatic variability and fungal diversity profiles identified indoors, and further connections to explore in future research were the relatability of CFU levels with the amount of settled dust and extractable DNA on surfaces. Interesting patterns, concerning fungal loads, community compositions, microclimatic and other site-specific factors, were found in the comparison between the sites. Their geographic location and nearby outdoor surroundings as well as indoor (micro)climatic fluctuations were shown to be key factors. However, there is currently no indication of a “museum fungal fingerprint” to be found. Thus, the opposite inclination is considered: the presence of all the major genera that contain species that are potentially harmful to collections must be assumed. This, in turn, implies that a similar risk adheres to all indoor spaces with heightened CFU levels, provided there is no indoor source. As was discovered in our data, even the seemingly most airtight indoor depot rooms with full HVAC control react to developments of fungal loads in outdoor air. Focusing more on cleaning intervals and maintenance of ventilation systems, paying special attention to filter materials, rather than relying solely on fixed climatic threshold values, can therefore prove to be very effective in the reduction in potential mold growth risks within these museum depots.

## Figures and Tables

**Table 1:** Outdoor and indoor climate: annual averages of temperature (T) and relative humidity (RH) outside the buildings and inside the investigated depots, calculated from the first year. Level of active climate control (ACC) available: none, dehumidification (D), heating (H), full (HVAC).

Location		D1	D2	D3	D4	D5	D6	D7	D8	D9
Indoor ACC		none	HVAC	HVAC	none/(D)	none/(H)	HVAC	none/(D)	HVAC	HVAC
**Average** **T (°C)**	Indoor	15.3 ± 4.6	21.1 ± 0.7	20.1 ± 0.3	18.0 ± 4.7	20.0 ± 3.2	19.9 ± 0.3	23.7 ± 1.0	22.1 ± 1.2	19.3 ± 0.5
	Outdoor	11.6 ± 7.0	11.6 ± 7.0	12.5 ± 7.1	11.6 ± 6.8	11.6 ± 6.8	12.7 ± 7.0	11.6 ± 6.8	13.8 ± 7.1	13.8 ± 7.1
**Average RH (%)**	Indoor	54.9 ± 3.5	48.9 ± 2.0	52.4 ± 1.2	52.1 ± 2.5	43.2 ± 3.5	52.7 ± 1.3	31.5 ± 3.5	50.9 ± 4.3	52.7 ± 2.6
	Outdoor	70.0 ± 12.9	70.0 ± 12.9	62.7 ± 12.8	67.8 ± 12.4	67.8 ± 12.4	62.6 ± 12.4	67.8 ± 12.4	57.5 ± 11.7	57.5 ± 11.7

## Data Availability

Publicly available data are given as URLs; metagenomic data are available in the NCBI public database (https://www.ncbi.nlm.nih.gov/, NCBI BioProject accession number PRJNA904284). Other data collected during the project are available on application to K.D.

## Supplementary Materials

The following supporting information can be downloaded at https://www.mdpi.com/article/10.3390/jof11070478/s1. Table S1: Table with details on all investigated buildings and rooms, including positions of thermohygrometric sensors, materials within rooms, cleaning and visitor frequencies. Table S2: Outdoor weather conditions, temperature (T) and relative humidity (RH) during winter and summer sampling days in all four seasons (W1, S1, W2, S2). Table S3: QC Filtered Reads and assigned reads after classification with Emu. Figure S1: Dendrogram showing clustering analysis results between all indoor climate (T and RH) average values and standard deviations for all nine museum depots.
