# Supplementary material for: Monitoring of Fungal Diversity and Microclimate in Nine Different Museum Depots
Source: J Fungi (Basel). 2025 Jun 24;11(7):478. doi: 10.3390/jof11070478 (PMC12295070; doi:10.3390/jof11070478)
Supplement: Supplementary file 1 [file jof-11-00478-s001.zip › jof-3672109-supplementary.pdf]

Everything, everywhere, anytime? Fungal Diversity and Microclimate in nine different Museum Depots.

Katharina Derksen [1,*], Peter Brimblecombe [2,3], Guadalupe Piñar [1], Monika Waldherr [4], Alexandra Graf [4], Pascal Querner [5,6] and Katja Sterflinger [1,*]

Supplementary Materials

Table S1: Table with details on all investigated buildings and rooms, including positions of thermohygrometric sensors, materials within rooms, cleaning and visitor frequencies.

Table S2: Outdoor weather conditions, temperature (T) and relative humidity (RH) during winter and summer sampling days in all four seasons (W1, S1, W2, S2).

Table S3: QC Filtered Reads and assigned reads after classification with Emu.

Figure S1: Dendrogram showing clustering analysis results between all indoor climate (T and RH) average values and standard deviations for all nine museum depots.

Table S1:

Location	Sensor	Floor level	Sensor position	Height (cm)	HVAC	Windows	Cleaning	Visitors	Floor Material	Type of collection
D1	HA001	0	room center, shelf	180	no	no (but airy space)	1 x per year, dry	1 per week, 60 min	asphalt	diverse, mostly wood, metal
D1	HA002	0	room center, floor	0						
D1	HA003	0	wall	180						
D1	HA003e	0	floor	0						
D1	HA004	0	wall	180						
D1	HA004e	0	floor	0						
D2b	SP002	2	floor, next to door	0	yes, full	2x, all closed	1 x per year, dry	1 per day, 60 min	*Sika-floor cure hard 24*	Paper
D2a	SP004	0	floor, behind painting racks	0	yes, full, +wall heating	no	1 x per year, dry	1 per day, 30 min	linoleum	Paintings
D3	KT001	2	room center, concrete pillar	180	yes, full	no	1 x per year, dry	1 per day, 60 min	concrete	diverse
D3	KT002	2	room center, concrete pillar	180						
D3	KT003	2	on S-wall	180						
D3	KT003e	2	S-wall, floor	0						
D3	KT004	2	on N-wall	180						
D3	KT004e	2	N-wall, floor	0						
D4a	WB005	1	floor	0	no, but dehumidification	10x, all closed	every day, dry, oil	1 per day, 30 min	bitumen	leather, textile, wood, metal
D4a	WB007	1	shelf (object mount)	180						
D4a	WB008	1	room center, floor	0						
D4b	WB001	0	right wall	180	no, but dehumidification	10x, all closed	every day, dry, oil	1 per day, 30 min	wood	wood, metal, textile
D4b	WB001e	0	floor	0						
D4b	WB002	0	left wall	180						
D4b	WB002e	0	floor	0						
D4b	WB003	0	floor next to door	0						
D5a	SB005	1	room center	160	no (adjacent rooms heated)	3x, all closed	every 2 years, dry, vac.	1, 2x per week, 10 min	wood, new	diverse (ceramic, metal, textile...)
D5a	SB005e	1	floor	0						
D5b	SB007	1	wall	180	no (adjacent rooms heated)	1x, closed	every 2 years, dry, vac.	1, 2x per week, 10 min	wood, new	diverse
D5b	SB007e	1	floor	0						

Table S1 continues on next page.

Location	Sensor	Floor level	Sensor position	Height (cm)	HVAC	Windows	Cleaning	Visitors	Floor material	Type of collection
D6a	EM001	0	room center	200	yes, full	no	1 x per year, dry	1 per day, 60 min	epoxy resin	diverse
D6a	EM002	0	wall next to door	180						
D6a	EM002e	0	floor	0						
D6b	EM003	0	room center	200	yes, full	no	1 x per year, dry	1 per day, 60 min	epoxy resin	paintings
D6b	EM004	0	wall next to door	180						
D6b	EM004e	0	floor	0						
D6b	EM005	0	next to shelf with paper	180						
D6b	EM005e	0	floor	0						
D6b	EM006	0	between paintings	180						
D7	TM006	-1	wall	180	no, but dehumidification	no	1 x per year, dry	1 per day, for 60 min	epoxy resin	archive, mainly paper
D7	TM006e	-1	floor	0						
D8a	WM001	-1	shelf	180	yes, full	1x, closed	1x per year, dry, vac., floors wet	1, 2x per week, 10 min	bitumen	diverse, wood, textile
D8a	WM001e	-1	floor	0						
D8b	WM002	-1	shelf	200	yes, full	2x, all closed	1x per year, dry, vac., floors wet	1, 2x per week, 10 min	bitumen	diverse, ceramics
D8b	WM002e	-1	floor	0						
D8c	WM003	-1	shelf	200	yes, full	1x, closed	1x per year, dry, vac., floors wet	1, 2x per week, 10 min	bitumen	diverse, wood, textile
D8c	WM003e	-1	floor	0						
D8d	WM006	-1	shelf	200	yes, full	3x, all closed	1x per year, dry, vac., floors wet	1, 2x per week, 10 min	bitumen	diverse, mainly leather
D8d	WM006e	-1	floor	0						
D9	EA001	1	floor	0	yes (set to 20 °C, 50 % RH)	5x, all closed	every 2 years, dry, vac.	1, 2x per week for 10 min	linoleum	archive, mainly paper
D9	EA002	1	room center, shelf	150						
D9	EA003	1	outside wall	150						
D9	EA003e	1	floor	0						
D9	EA004	1	inside wall	150						
D9	EA004e	1	floor	0						

Table S2:

Location	Conditions	T [°C]	RH [%]
**D1-W1**	sunny, light wind	2°C	77%
**D1-S1**	cloudy, light rain, no wind	12°C	95%
**D1-W2**	sunny, light wind	-1°C	72%
**D1-S2**	sunny, breeze	25°C	68%
**D2-W1**	sunny, light wind	2°C	77%
**D2-S1**	cloudy, light rain, no wind	12°C	95%
**D2-W2**	sunny, light wind	-1°C	72%
**D2-S2**	sunny, breeze	25°C	68%
**D3-W1**	cloudy, light rain	-1°C	86%
**D3-S1**	sun/clouds, light wind	17°C	73%
**D3-W2**	sun/clouds, breeze	7°C	74%
**D3-S2**	sunny, breeze	20°C	73%
**D4-W1**	sunny, windy	6°C	68%
**D4-S1**	sunny, no wind	27°C	48%
**D4-W2**	sunny, windy	6°C	70%
**D4-S2**	cloudy, breeze	17°C	70%
**D5-W1**	sunny, windy	6°C	68%
**D5-S1**	sunny, no wind	27°C	48%
**D5-W2**	clouds/sun, light wind	1°C	62%
**D5-S2**	cloudy/light rain, no wind	20°C	85%
**D6-W1**	cloudy/light rain, windy	4°C	63%
**D6-S1**	sun/clouds, light wind	24°C	50%
**D6-W2**	cloudy, snow on ground	-4°C	74%
**D6-S2**	sunny, breeze	26°C	35%
**D7-W1**	sun/clouds, light wind	5°C	70%
**D7-S1**	sun/clouds, no wind	25°C	55%
**D7-W2**	cloudy, breeze	2°C	69%
**D7-S2**	cloudy, light wind	17°C	87%
**D8-W1**	cloudy	0°C	86%
**D8-S1**	sun/clouds, no wind	23°C	78%
**D8-W2**	cloudy, light wind	3°C	82%
**D8-S2**	clouds/sun, breeze	24°C	35%
**D9-W1**	sunny, windy	6°C	58%
**D9-S1**	sun/clouds, breeze	23°C	59%
**D9-W2**	cloudy, no wind	-3°C	68%
**D9-S2**	sunny, windy	33°C	39%

Table S3:

Barcode	Sample	Total Bases	Reads	Median Length	Median QS	Assigned Reads
BC01	D8	2,121,181,975	4,148,105	499.0	19.6	4,017,352
BC02	D4	3,402,917,370	6,752,533	488.0	20.2	6,635,685
BC03	D5	1,920,996,932	3,676,263	527.0	19.7	3,483,469
BC04	D7	2,350,175,838	4,564,467	514.0	18.6	4,283,216
BC05	D9	442,109,999	863,541	505.0	18.4	806,698
BC06	D3	2,027,789,814	4,436,847	440.0	18.7	4,417,925
BC07	D2	1,615,187,529	3,365,303	456.0	18.6	3,213,608
BC08	D1	1,134,089,629	2,370,250	473.0	18.1	2,349,607

Figure S1:

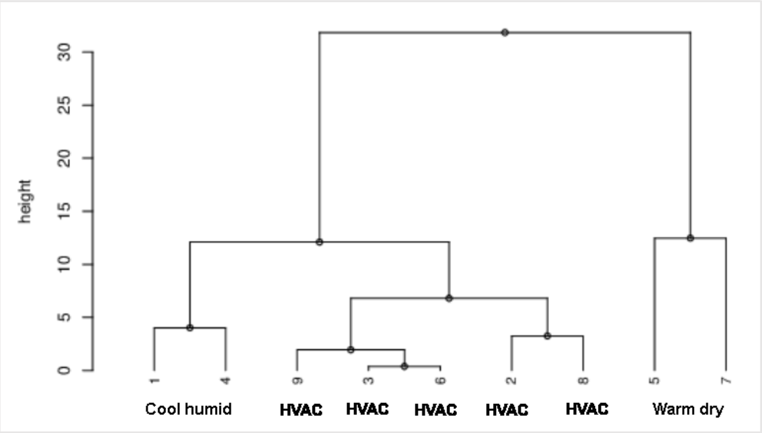
height
30
25
20
15
10
5
0
1
4
9
3
6
2
8
5
7
Cool humid
HVAC
HVAC
HVAC
HVAC
HVAC
Warm dry
